# No difference in clinical outcomes in robotic-assisted vs. computer-navigated total hip arthroplasty

**DOI:** 10.1186/s42836-025-00306-1

**Published:** 2025-05-01

**Authors:** Haroun Haque, Ameer Tabbaa, Matthew Johnson, Lydia Fu, Afshin Razi, Matthew L. Magruder

**Affiliations:** 1 College of Medicine, SUNY Downstate Health Sciences University, Brooklyn, NY 11203 USA; 2 Department of Orthopaedic Surgery, Maimonides Medical Center, Brooklyn, NY 11219 USA

**Keywords:** Robotic-assisted, Computer-navigated, Total hip arthroplasty, Clinical outcomes, Implant-related complications

## Abstract

**Background:**

Robotic-assisted (RA) and computer-navigated (CN) total hip arthroplasty (THA) are increasingly performed, but prior studies comparing the two techniques and their outcomes were limited. This study aimed to compare clinical outcomes and costs of receiving THA using RA and CN technology.

**Methods:**

We conducted a retrospective cohort study using a nationwide administrative database from January 1, 2010, to October 31, 2022. The study included patients undergoing THA either via RA (*n* = 4,473) or CN (*n* = 4,473) technology. Subjects were matched for age and pertinent comorbidities. Clinical outcomes evaluated included emergency department visits and readmissions within 90 days of surgery, lengths of stay, and implant-related complications within 90 days and 2 years of surgery. Costs were analyzed on the day of surgery and within a 90-day global period. Statistical analysis was performed using multivariate logistic regression analysis with a *P* < 0.01 considered statistically significant.

**Results:**

There were no significant differences between the RA and CN cohort in ED visits or readmission within 90 days of surgery or in lengths of stay. Similarly, no differences were found in any of the implant-related complications at 90 days or 2 years following surgery. Same-day mean reimbursement for RA-THA was higher than for CN-THA ($4,472.23 *vs*. $3,890.61; *P* < 0.01). 90-day reimbursement did not differ significantly.

**Conclusion:**

We demonstrated that readmission, ED visits, lengths of stay, and short-term implant-related outcomes did not differ between RA and CN-THA cohorts. Further studies are needed to explore the long-term benefits and cost implications of RA-THA.

## Introduction

The use of technology to assist in performing total hip arthroplasty (THA) has increased in recent years, consisting of robotic-assisted (RA) and computer-navigated (CN) technologies [1, 2]. Both RA- and CN-THA systems are designed to adapt to the patient's anatomy to accurately map the femur and acetabulum in space. RA-THA utilizes a haptically controlled robotic arm, which the surgeon manipulates with some level of control from the system, thereby reducing the risk of error and enhancing precision in implant placement [3]. On the other hand, CN-THA provides real-time guidance through computer algorithms and imaging but leaves the manual task of implant placement entirely to the surgeon, offering intraoperative feedback on alignment and positioning [4]. From 2005 to 2018, the number of CN-THA procedures in the United States increased from 0.1% to 1.9%^2^. From 2008 to 2018, the number of RA-THA procedures rose from < 0.1% to 2.1%^2^. The increasing prevalence of both these technologies underscores the importance of their study and comparative evaluation.

The literature regarding postoperative complications of RA and CN-THA is mixed. Prior reviews have demonstrated that RA-THA may be linked to greater dislocation rates compared to CN-THA [5]. RA-THA was not shown to be linked to a statistically significant increase in revision rates when compared to CN procedures. Other studies found no significant differences in surgical outcomes between RA and CN techniques, questioning the clinical relevance of the reported statistical differences [6]. Given the mixed evidence and uncertainty regarding the advantages and disadvantages of RA *vs*. CN-THA, there is a need for further investigation of infection rates, dislocation rates, readmission rates, and the cost for each of these procedures.

In this retrospective cohort study of an administrative database, we aimed to evaluate the 90-day and 2-year clinical outcomes of RA and CN-THA, including emergency department visits, readmissions, lengths of stay, implant-related outcomes, and costs.

## Methods

### Database and patient selection

A retrospective query was performed using the administrative claim database PearlDiver (PearlDiver Technologies, Fort Wayne, IN, USA) from January 1, 2010, to October 31, 2022. This database adheres to the Health Information Portability and Affordability Act and has been widely utilized for orthopedic surgery research in the past [7–9]. As this dataset consists of deidentified patient information, our study was exempt from approval from our institution's internal review board.

The database was queried using the International Classification of Disease (ICD), the Ninth and Tenth Revisions, and Current Procedural Terminology (CPT) Codes. The study included patients undergoing THA either through robotic-assistance or computer-navigation, identified against specific ICD-9/10 and CPT codes that were linked to each type of procedure within the Pearldiver database. For RA procedures, procedural codes were ICD-10-P-8E0YXCZ and ICD-10-P-8E0Y0CZ. For CN procedures, procedural codes were ICD-10-P-8E0YXBZ, ICD-10-P-8E0YXBF, ICD-10-P-8E0YXBF, and ICD-10-P-8E0YXBG. Subjects were matched for age and comorbidities, including obesity, renal failure, peripheral vascular disease (PVD), rheumatoid arthritis, depression, hypertension, alcohol abuse, and diabetes. Subjects were matched for Elixhauser Comorbidity Index (ECI). Matching was successful, producing 4,473 patients in the RA cohort and 4,473 patients in the CN cohort (Table 1).

**Table 1:** Propensity score matching characteristics demonstrated no differences in age, sex, medical comorbidities, or Elixhauser Comorbidity Index (ECI)

Demographics	RA (*n* = 4473)		CN (*n* = 4473)		*P*-Value
Age	*n*	%	*n*	%	0.99
30–34	13	0.29	13	0.29	
35–39	23	0.51	23	0.51	
40–44	56	1.25	56	1.25	
45–49	141	3.15	141	3.15	
50–54	290	6.48	290	6.48	
55–59	574	12.83	574	12.83	
60–64	830	18.56	830	18.56	
65–69	838	18.73	838	18.73	
70–74	777	17.37	777	17.37	
75–79	654	14.62	654	14.62	
80 +	270	6.04	270	6.04	
Sex					0.99
Female	2519	56.32	2519	56.32	
Male	1954	43.68	1954	43.68	
Medical comorbidities					0.99
Obesity	2254	50.39	2254	50.39	
Renal failure	492	11.00	492	11.00	
PVD	845	18.89	845	18.89	
Rheumatoid arthritis	76	1.70	76	1.70	
Depression	1391	31.10	1391	31.10	
Hypertension	3417	76.39	3417	76.39	
Alcohol abuse	146	3.26	146	3.26	
Diabetes	1427	31.9	1427	31.9	
ECI					0.99
1	213	4.76	213	4.76	
2	486	10.87	486	10.87	
3	685	15.31	685	15.31	
4	695	15.54	695	15.54	
5	610	13.64	610	13.64	
6	480	10.73	480	10.73	
7	388	8.67	388	8.67	
8	283	6.33	283	6.33	
9	198	4.43	198	4.43	
10	149	3.33	149	3.33	
11	111	2.48	111	2.48	
12	67	1.50	67	1.50	
13	41	0.92	41	0.92	
14	30	0.67	30	0.67	
15	21	0.47	21	0.47	
16	11	0.25	11	0.25	
17	0	0.00	0	0.00	

*RA* Robotic-assisted, *CN* Computer-navigated, *PVD* Peripheral vascular disease

### Study outcomes and data analyses

Outcomes of interest included 90-day emergency department (ED) visits, readmissions, lengths of stay, 90-day and 2-year rates of implant-related complications, and same-day surgical and 90-day global episode of care reimbursements. Implant-related complications included rates of dislocations, revision surgery, aseptic loosening, fractured prosthetic implant, periprosthetic fracture (PPFx), superficial surgical site infection (SSI), and deep prosthetic joint infection (PJI). Multivariate logistical regressions were used to calculate odds ratios (ORs), 95% confidence intervals, and *P* values. A *P*-value of < 0.01 was used as the significance threshold to minimize type 1 error. All statistical analyses were performed using R Statistical Software (version 3.3.3; R Foundation for Statistical Computing, Vienna, Austria).

## Results

Readmission rates did not differ significantly between groups (6.68 vs. 6.26%; OR 1.22; *P* = 0.185). ED visits within 90 days were similar for both groups (3.24 vs. 2.1%; OR 1.76; *P* = 0.09). Lengths of stay were also comparable for each group (1.888 vs 1.855 days; *P* = 0.087) (Table 2).

**Table 2:** Comparison of robotic-assisted (RA) vs. computer-navigated (CN) THA 90-day readmissions, ED visits, and lengths of stay

Outcome	RA (*n* = 4,473)	%	CN (*n* = 4,473)	%	OR	95% CI	*P*-value
Readmissions	299	6.68%	280	6.26%	1.22	0.81–1.83	0.185
ED Visits	145	3.24%	94	2.10%	1.76	0.87–3.24	0.0896
Lengths of stay (mean)	1.88		1.855				0.0873

*OR* Odds ratio, *CI* Confidence interval

Differences in 90-day dislocation rates were not statistically significant (0.56 vs. 0.76%; *P* = 0.978). Differences in 90-day revisions were not found to be statistically significant (0 vs 0%; *P* = 1). 90-day aseptic loosening rates (0.25 vs. 0.27%;* P* = 0.992) were similar for both groups. 90-day fractured prosthetic implant rates did not differ between groups (0 vs. 0%; *P* = 1). 90-day periprosthetic fracture (PPFx) rates (0.83 vs. 0.585; OR 0.89 *P* = 0.877) were comparable across groups. 90-day surgical site infections (SSI) did not differ significantly between groups (0.31 vs. 0.27%; OR 0.87; *P* = 0.896). Periprosthetic joint infections (0.65 vs. 0.65%; OR 0.92; *P* = 0.90) were similar across both groups (Table 3).

**Table 3:** Comparison of robotic-assisted (RA) vs. computer-navigated (CN) THA 90-day and 2-year implant-related outcomes

Outcome	RA (*n* = 4,473)	%	CN (*n* = 4,473)	%	Odds ratio (OR)	95% CI	*P*-value
**Implant complications (within 90 days)**							
Dislocations	25	0.56%	34	0.76%	N/A	N/A	0.979
Revisions	0	0.00%	0	0.00%	1.00	0.00–∞	1.000
Aseptic Loosening	11	0.25%	12	0.27%	N/A	N/A–N/A	0.992
Fractured Prosthetic Implant	0	0.00%	0	0.00%	N/A	N/A	1.000
PPFx	37	0.83%	26	0.58%	0.89	0.14–3.13	0.877
SSI	14	0.31%	12	0.27%	0.87	0.05–4.80	0.896
PJI	29	0.65%	29	0.65%	0.92	0.21–2.78	0.900
**Implant complications (within 2 years)**							
Dislocations	58	1.30%	53	1.18%	0.54	0.13–1.53	0.313
Revisions	16	0.36%	19	0.42%	0.84	0.43–1.66	0.735
Aseptic loosening	33	0.74%	34	0.76%	1.20	0.28–3.50	0.766
Fractured prosthetic implant	0	0.00%	0	0.00%	N/A	N/A	1.000
PPFx	54	1.21%	40	0.89%	1.61	0.54–3.93	0.337
SSI	26	0.58%	25	0.56%	0.82	0.13–2.91	0.789
PJI	55	1.23%	46	1.03%	1.19	0.40–2.85	0.723

*OR* Odds ratio, *CI* Confidence interval, *PPFx* Periprosthetic fracture, *SSI* Surgical site infection, *PJI* Periprosthetic joint infection. Definitions: *N/A* Not Applicable. Odds ratio and confidence intervals were not calculable due to zero event rates in both groups or insufficient data for statistical analysis. < 11: Event rates less than 11 are not reported due to database privacy policies to prevent potential identification of patients

There was no significant difference in 2-year dislocation rates between the two groups (1.30 vs 1.18%; OR 0.54; *P* = 0.313). Differences in 2-year revision rates were also not found to be statistically significant (0.36 vs. 0.42%; OR 0.84 *P* = 0.735). 2-year aseptic loosening rates (0.74 vs 0.76%; OR 1.2; *P* = 0.766) did not differ significantly between groups. 2-year fractured prosthetic implant rates (0 vs. 0%; *P* = 1) did not differ between groups. 2-year periprosthetic fracture (PPFx) rates did not differ between groups (1.21 vs. 0.89%; OR 1.61; *P* = 0.337). Surgical site infection (SSI) rates did not differ between groups (0.58 vs. 0.56% OR 0.82; *P* = 0.789. Periprosthetic joint infection (PJI) rates did not differ between groups (1.23 vs. 1.03%; OR 1.19; *P* = 0.723) (Table 3).

There were statistically significant differences in the mean same-day reimbursement ($4,472.23 vs $3,890.61; *P* = 0.0046) (Table 4). The 90-day mean reimbursement did not differ significantly between the RA and CN groups ($7,332.27 vs. $6,926.61; *P* = 0.3547) (Table 4).

**Table 4:** Mean reimbursement for robotic-assisted (RA) and computer-navigated (CN) THA

Costs	RA (*n* = 4473)	CN (*n* = 4473)	*P*-value
Same day mean reimbursement	$4,472.23	$3,890.61	0.004628
90 days mean reimbursement	$7,332.27	$6,926.61	0.3547

## Discussion

In this investigation, we demonstrated that patients who underwent RA-THA did not have significantly different 90-day ED visits, 90-day readmissions, lengths of stay, 90-day implant-related complications, or 2-year implant-related complications. Furthermore, while the same-day reimbursements were high for RA-THA, those differences did not extend to 90 days following surgery. This suggests that, despite the known increased cost associated with robotic technology, there may not be any additional benefit to the patient nor additional reimbursement to the surgeon at this time.

Direct comparisons of RA and CN-THA outcomes in the literature are limited. Three studies have attempted to compare clinical outcomes between the two techniques. The first was a retrospective study using a single institution database, which included 896 patients who underwent CN-THA, 135 patients who underwent RA-THA, and 929 patients who underwent conventional-THA. When ANCOVA analysis was conducted across groups, there was no difference in 90-day readmissions (*P* = 0.792) or 90-day revisions (*P* = 0.839) among the three groups. There was, however, a statistically significant reduction in surgical time and length of stay for CN-THA (*P* > 0.001). It is important to note that this study did not directly compare RA and CN-THA outcomes but instead performed analysis across three groups, which limits the ability to make definitive conclusions about the relative effectiveness of the two techniques [6].

A second investigation conducted a metanalysis of randomized controlled trials (RCTs) comparing studies that evaluated either RA-THA or CN-THA to conventional-THA, which may serve as an indirect method of comparing the two techniques. Five RCTs comparing RA-THA to conventional-THA and 7 RCTs comparing CN-THA to conventional-THA were evaluated. This investigation reported that revision rates for RA and CN-THA did not differ significantly (*P* = 0.11). Dislocation rates across RA, CN, and conventional-THA, however, were found to be significantly higher for RA-THA (4.4%) when compared to CN (0.34%) and conventional-THA (0.7%) (*P* < 0.001) [5]. The differences in all-cause complications (CN: 1.7%, RA: 16.2%, Conventional: 6.6%) were not found to be statistically significant [5].

The literature has produced mixed results regarding the comparative efficacy of RA and CN-THA in terms of clinical outcomes. Some studies suggest that RA-THA may be linked to higher rates of dislocation compared to CN, while revision rates appear to be similar across both procedures [5]. Other studies indicate that there are no significant differences in key clinical outcomes between these two approaches [6]. The investigation that identified increased rates of dislocations for RA-THA did not directly compare the two techniques, and therefore, results may have been influenced by underlying differences in patient populations [5]. Additionally, this study included RA-THA cases as early as 2003, and its findings may not reflect outcomes with current robotic technology.

A recent single-institution study comparing RA-THA, CN-THA, and conventional THA found no statistically significant differences in rates of intraoperative fracture, dislocation, or infection [10]. However, the study was limited by its single-center design, which may not account for variations in surgical technique and patient populations across institutions. Additionally, the inclusion of conventional THA as a third comparison group introduced greater variability, making it more difficult to isolate the effects of RA vs. CN techniques. Our results are consistent with the literature, suggesting no significant difference in clinical outcomes between RA and CN-THA.

Despite no clear improvement in clinical complication rates with RA-THA, there remains a possibility that functional or patient-reported outcome measures (PROMs) may differ between techniques. In one retrospective investigation, RA-THA propensity scores were matched to those of patients who received conventional THA procedures and found significant improvements with RA-THA. The authors reported significantly higher Oxford Hip Score (OHS) and Forgotten Joint Score (FJS) at 1 year following surgery for the RA cohort [11]. Another retrospective study demonstrated improved PROMs for both CN and RA-THA when compared to conventional THA, with improvements being noted in a greater percentage of the RA-studies (60% vs. 33.3%). However, the authors reported that these differences were not clinically significant in any of the studies comparing RA to conventional THA and were only clinically significant in 1 of 6 (16.7%) of studies comparing CN to conventional THA [12]. These findings align with our results, further reinforcing the notion that while advanced technologies may offer some technical advantages, they do not consistently translate into clinically meaningful improvements for patients. It is important to note that no studies have directly compared these functional outcomes for RA and CN-THA to one another. Further investigations are warranted to determine whether there is a real difference in functional outcomes for patients receiving RA vs. CN-THA.

The lack of significant differences in clinical outcomes between RA and CN-THA may be attributed to the fact that much of the benefit in these technologies comes from the addition of computer-navigation. Both methods involve precise calibration and guidance, ensuring accurate implant placement. The addition of a robotic-arm providing active or semi-active control in RA-THA, while technologically advanced, may not provide substantial additional benefits for patient outcomes beyond what is already achieved through navigation alone.

Our study found no significant difference in 90-day reimbursement between the CN and RA cohorts. Ninety-day reimbursement represents the total payment received within the 90-day global period and is the most clinically relevant metric, as it encompasses key reimbursements for nearly all payers. While 90-day reimbursement is an important financial metric for hospital systems, it does not fully capture long-term cost-effectiveness beyond the global period, such as extended rehabilitation costs, indirect societal costs related to return to work, or downstream healthcare utilization.

Given the higher initial costs associated with the robotic technology, there may be no additional benefits for the surgeon or the hospital system. Without a significant clinical or financial incentive, the choice between RA and CN-THA may come down to the surgeon’s preference and institutional resources rather than the presumed technological superiority of one approach over the other.

This study was limited by its nature as a national claims database study, relying on the accuracy of accurate coding of all medical procedures and complications by physicians. However, Medicare claims data undergo adjudication, regular audits, and internal reviews, with providers required to contract with independent third parties for annual validity and reliability assessments [13]. Additionally, the retrospective nature of the analysis limited our ability to control for all potential confounders. Furthermore, there are many different RA and CN technologies, which are not all created equal. We were not able to conduct sub-analyses based on RA or CN characteristics (e.g., image-based vs. imageless). It is possible that clinical outcomes may be improved in the image-based technologies, for example. Furthermore, we were not able to account for important differences in surgical technique (e.g., anterior vs. posterior approach) based on insurance codes, although these factors have previously been demonstrated to be clinically relevant [14]. While this study did not find significant differences in the primary outcomes assessed, future research should continue to explore other potential benefits of these technologies, such as long-term functional outcomes, patient-reported satisfaction, and detailed cost-effectiveness. Additionally, investigations into specific patient subgroups, such as those with complex anatomical variations or higher risk profiles, may uncover differential benefits that were not apparent in the broader study population.

## Conclusion

Presented here is a retrospective analysis of an administrative database that demonstrated that both RA and CN technologies used during THA yielded comparable readmission, ED visits, implant-related outcomes, and 90-day episodes of care reimbursements. Further research is warranted to explore additional dimensions of clinical and economic impact, ultimately guiding optimal utilization of these technologies in orthopedic surgery.

## Data Availability

All data were generated using the administrative claim database PearlDiver (PearlDiver Technologies, Fort Wayne, IN).

## Abbreviations

CN: Computer-Navigated
CPT: Current Procedural Terminology
ECI: Elixhauser Comorbidity Index
ED: Emergency Department
FJS: Forgotten Joint Score
ICD: International Classification of Disease
OHS: Oxford Hip Score
OR: Odds Ratio
PJI: Prosthetic Joint Infection
PPFx: Periprosthetic Fracture
PROM: Patient-Reported Outcome Measure
PVD: Peripheral Vascular Disease
RA: Robotic-Assisted
RCT: Randomized Controlled Trial
SSI: Superficial Surgical Site Infection
THA: Total Hip Arthroplasty
